# Hyponatremia and the risk of kidney stones: A matched case-control study in a large U.S. health system

**DOI:** 10.1371/journal.pone.0203942

**Published:** 2018-09-21

**Authors:** Naoto Tominaga, Stephen J. Fernandez, Mihriye Mete, Nawar M. Shara, Joseph G. Verbalis

**Affiliations:** 1 Division of Endocrinology and Metabolism, Georgetown University Medical Center, Washington, DC, United States of America; 2 Department of Biostatistics and Biomedical Informatics, MedStar Health Research Institute, Hyattsville, MD, United States of America; Medical College of Wisconsin, UNITED STATES

## Abstract

Kidney stones impose a large and increasing public health burden. Previous studies showed that hyponatremia is associated with an increased risk of osteoporosis and bone fractures, which are also known to be associated with kidney stones. However, the relation between hyponatremia and kidney stones is not known. To assess the relation between hyponatremia and kidney stones, we designed a matched case-control study by using the electronic health records of the MedStar Health system with more than 3.4 million unique patient records as of March 2016. Data were extracted for clinical factors of patients with kidney stones (cases) and those without kidney stones (controls). Cases (n = 20,199) and controls (n = 20,199) were matched at a 1:1 ratio for age, sex, race, and the duration of encounter window. Case and control exposures for each of the hyponatremia variables were defined by serum sodium laboratory measurements reported within the encounter windows, and divided into 3 categories: prior hyponatremia, recent hyponatremia, and persistent hyponatremia. In the final conditional logistic models adjusted for potential confounders, the risk of kidney stones significantly increased in both recent and persistent hyponatremia categories: prior hyponatremia odds ratio (OR) 0.93 (95% confidence interval [CI], 0.86–1.00); recent hyponatremia OR 2.02 (95% CI, 1.76–2.32); persistent hyponatremia OR 6.25 (95% CI, 3.27–11.96). In conclusion, chronic persistent hyponatremia is a significant and clinically important risk factor for kidney stones in patients in the U.S.

## Introduction

Approximately 11% of men and 7% of women in the United States (U.S.) will develop at least one kidney stone (KS) during their lifetime [1]. The annual expenditure for KS in the U.S. is estimated to exceed $10 billion [2], thus the economic and social burden of KS is large. KS has been reported as a potential risk factor for osteoporotic fractures [3], and a previous study showed that the prevalence of low bone mineral density (BMD) in some skeletal sites is higher in patients with KS [4].

Hyponatremia, the most common electrolyte abnormality in clinical practice, is known as a well-established risk factor for morbidity and mortality across a wide variety of underlying diseases [5], and correcting hyponatremia has been associated with decreased mortality [6]. Moreover, a recent study showed that hyponatremia is also associated with increased risks of osteoporosis and bone fractures [7]. A potential common link between hyponatremia and KS is increased urine calcium (Ca^2+^) excretion, which has been suggested in an animal model of hyponatremia-induced osteoporosis [8], and shown in a clinical research study of patients with the syndrome of inappropriate antidiuretic hormone secretion (SIADH) [9]. However, whether hyponatremia is correlated with the occurrence of KS has not been studied. The objective of this study was to evaluate whether there is an association between hyponatremia and KS in a large U.S. health system population.

## Methods

### Patient population and data source

The data for this study was obtained from the MedStar Health pooled patient electronic health records (EHR) database in Maryland, Virginia, and greater Washington, DC in the U.S. De-identified patient data were extracted using the Explorys tool [10]. There were more than 3.4 million unique patient records in the database available for query at the start of the study. Our dataset includes patient EHR for the period 1998–2016, but the subset of patients with observations prior to 2002 is limited due to the official implementation of EHR system at MedStar Health institutions being in early 2000s.

This study was conducted in accordance with the Declaration of Helsinki, and was approved by the Institutional Review Board (IRB) at the MedStar Health Research Institute before starting the study (IRB approval number 2016–039); the requirement for informed consent was waived due to the de-identified nature of the analyses.

### Study design and procedures

In order to investigate the association between hyponatremia and KS, we designed a matched case-control study. Patients with KS were selected as cases and had to have at least one diagnosis of KS as defined by International Classification of Diseases ninth revision codes (ICD-9) for nephrolithiasis (592.0, 592.1, 592.9, 594, 594.1, 594.2, 594.8, and 594.9). In Explorys, ICD codes were automatically converted into the Systematized Nomenclature of Medicine (SNOMED).

Cases and controls with uric acid nephrolithiasis, diabetes mellitus, no serum sodium concentration ([Na^+^]) in the database, any concentration of serum [Na^+^] with a same-day glucose greater than 200 mg/dL, and cystic kidney disease by using ICD-9 codes, and intrinsic kidney diseases including chronic kidney disease (CKD) by using ICD-9 and/or -10 codes, were excluded from the analysis (S1 Table). The reasons for these exclusions were as follows: 1) the majority of KS are composed of Ca (CaKS) [11], and we hypothesize that hyponatremia may increase urine Ca^2+^ excretion as a result of bone resorption, 2) hyperglycemia is known to affect serum [Na^+^] concentration [12], and 3) we aimed to focus on systemic conditions that can influence the development of KS, separating them from renal comorbidities known to be associated with a high risk of KS.

**Fig 1:**
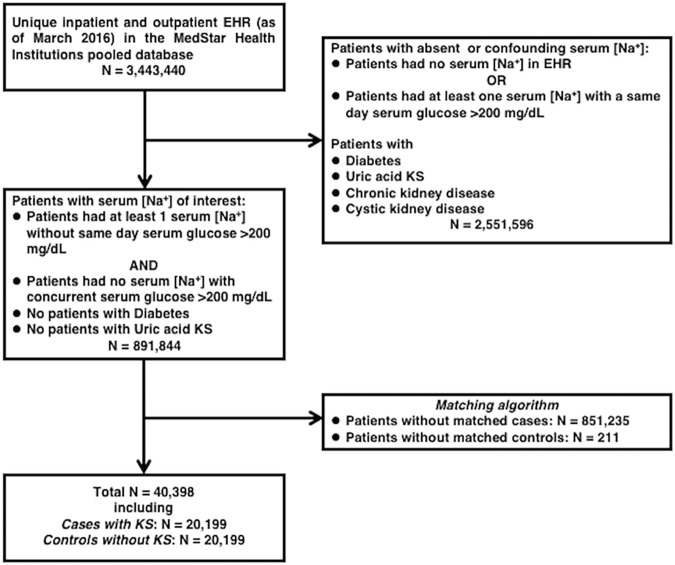
Strobe diagram. Abbreviations: EHR, electronic health records; KS, kidney stones; [Na^+^], sodium concentration.

One KS case was matched with one control without KS for the following covariables: age at first encounter (within 1 year), sex, race, and duration of the patient record (duration of encounter window) in the database (±1 month). Exact matching was performed with SAS 9.3 software using the Mayo Clinic gmatch general SAS macro. Based on the final number of matched cases used in the multivariable models, cases without matched controls and controls without matched cases were excluded. A strobe diagram of KS subject selection and matching is provided in Fig 1. An encounter window was defined for KS cases as the time between the date of the first encounter in the database and the date of the first KS diagnosis, which meant that patients who had KS at the time of the first encounter in the database were excluded. The encounter window of the matched cases defined the encounter windows for controls. A hypothetical last follow-up date for controls was calculated by adding the duration of the respective case’s encounter window to the control’s first encounter date.

Case and control exposures to the clinical variables of interest were defined by the documentation of at least one disease diagnosis, drug prescription, or behavioral diagnostic code during the encounter window by using ICD-9/10 revision codes (S2 Table). Variables of interest were hyponatremia, known risk-modified factors for KS (e.g., hypertension, obesity, dyslipidemia, gout, regional enteritis, ulcerative colitis, celiac disease, osteoporosis, hyperparathyroidism, hypercalcemia, acidosis, bariatric surgery and sarcoid as disease histories, calcium, estrogen, vitamin D, vitamin B6, vitamin C, thiazide, furosemide and topiramate as medication histories, and tobacco and alcohol use as behavioral histories) [13–16], and comorbidities frequently complicated by hyponatremia (heart failure and liver cirrhosis) [17].

**Fig 2:**
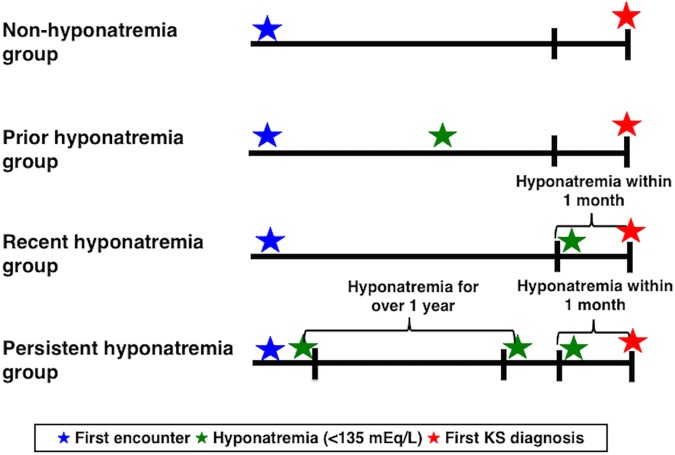
Definition of each hyponatremia group. The encounter windows for controls were defined by the encounter window of the matched cases before the first diagnosis of kidney stones (KS).

Case and control exposures for each hyponatremia category were defined by serum [Na^+^] laboratory measurements reported within the encounter windows as follows: 1) prior hyponatremia group, 2) recent hyponatremia group, and 3) persistent hyponatremia group. Exposure to prior hyponatremia was defined as having at least one serum [Na^+^] measurement of less than 135 mEq/L within the encounter window. Based on our previous study [7], each hyponatremia category was defined. Exposure to recent hyponatremia was defined as having at least one serum [Na^+^] of less than 135 mEq/L within 30 days before the end of the encounter window. Patients categorized as having only recent hyponatremia had a hyponatremic value within 30 days before the close of the encounter window, may or may not have had a second [Na^+^] on record, and had no hyponatremic value at an interval 1 year or greater from the close of the encounter window. Exposure to persistent hyponatremia was defined by meeting criteria for having both at least one serum [Na^+^] of less than 135 mEq/L within 30 days before the end of the encounter window (i.e., recent hyponatremia) and at least two serum [Na^+^] measurements of less than 135 mEq/L at least 1 year apart until 30 days before the end of the encounter window during the encounter window (Fig 2).

### Statistical analyses

All data were summarized using descriptive statistics such as means, standard deviations, frequencies and percentages. Unadjusted differences between cases and controls were tested using Mantel-Haenszel odds ratio test. Odds ratios (ORs) and 95% confidence intervals (CIs) were presented to show the changes in the odds of experiencing KS in response to clinical and demographic variables of interest. Multiple covariables were included in the final conditional logistic models, for matched case-control design to estimate the change in the risk of KS in the form of the OR, which measures the change in the odds of experiencing the outcome (KS), given the categories of an exposure variable. Statistical significance was defined as P < 0.05. Analyses were performed using SAS version 9.3 (SAS Institute) and Stata version 14 (StataCorp).

## Results

### Characteristics of KS cases and controls

In total, 20,199 KS cases and 20,199 controls were matched using a 1:1 ratio for age, sex, race, and the duration of encounter window (mean age, 43.6 ± 17.0 years, median age, 43 [31, 55], 48.7% female, 61.4% white, and mean encounter window 1304 ± 1506 days, median encounter window 813 [10, 2206] days, respectively), as shown in Fig 1 and Table 1.

**Table 1:** Characteristics of the study subjects and unadjusted odds ratios.

	Cases (N = 20,199)	Controls (N = 20,199)	OR (95% CI)	P value
**Sex, n, (%)**				
Female	9841 (48.7)	9841 (48.7)		
Male	10,358 (51.3)	10,358 (51.3)		
**Race, n, (%)**				
White	11,244 (61.4)	11,244 (61.4)		
African American	6238 (34.0)	6238 (34.0)		
Oriental/Other	693 (3.8)	690 (3.8)		
Asian/Pacific	12 (0.07)	9 (0.05)		
Native American or Alaskan	87 (0.47)	99 (0.54)		
Hispanic/Latino	29 (0.10)	25 (0.14)		
Multi-racial	19 (0.1)	19 (0.1)		
Unknown	1 (0.1)	0 (0)		
**Age at first encounter, years, mean (SD)/median [IQR]**	43.6 (17.0)/43 [31, 55]	43.6 (17.0)/43 [31, 55]		
**Duration of encounter window, days, mean (SD) /median [IQR]**	1304 (1506)/ 813 [10, 2206]	1304 (1506)/ 813 [10, 2206]		
**Medication history, n (%)**				
Calcium	176 (0.9)	151 (0.8)	1.17 (0.94–1.45)	0.17
Estrogen	312 (1.5)	353 (1.8)	0.87 (0.75–1.02)	0.10
Vitamin D	402 (2.0)	328 (1.6)	1.24 (1.07–1.45)	0.005
Vitamin B6	27 (0.1)	21 (0.1)	1.29 (0.73–2.27)	0.39
Vitamin C	130 (0.6)	113 (0.6)	1.15 (0.89–1.48)	0.28
Thiazide	807 (4.0)	667 (3.3)	1.23 (1.11–1.38)	0.0001
Furosemide	285 (1.4)	285 (1.4)	1.00 (0.85–1.18)	1.00
Topiramate	83 (0.4)	55 (0.3)	1.51 (1.07–2.12)	0.02
**Disease history, n (%)**				
Hypertension	5601 (27.7)	3998 (19.8)	1.75 (1.66–1.84)	<0.0001
Obesity	1930 (9.6)	1331 (6.6)	1.53 (1.42–1.65)	<0.0001
Dyslipidemia	3558 (17.6)	2719 (13.5)	1.47 (1.54–2.19)	<0.0001
Gout	360 (1.8)	199 (1.0)	1.84 (1.54–2.19)	<0.0001
Regional enteritis	224 (1.1)	56 (0.3)	4.06 (3.02–5.45)	<0.0001
Ulcerative colitis	92 (0.5)	72 (0.4)	1.28 (0.94–1.75)	<0.0001
Celiac disease	20 (0.1)	15 (0.1)	1.33 (0.68–2.60)	<0.0001
Osteoporosis	558 (2.8)	327 (1.6)	1.82 (1.57–2.10)	<0.0001
Hyperparathyroidism	136 (0.7)	33 (0.2)	4.22 (2.87–6.20)	<0.0001
Hypercalcemia	133 (0.7)	33 (0.2)	4.23 (2.86–6.25)	<0.0001
Acidosis	236 (1.2)	107 (0.5)	2.24 (1.78–2.82)	<0.0001
Bariatric surgery	89 (0.44)	34 (0.17)	2.62 (1.76–3.89)	<0.0001
Sarcoidosis	97 (0.5)	66 (0.3)	1.70 (1.22–2.36)	0.002
Liver cirrhosis	140 (0.7)	82 (0.4)	1.73 (1.31–2.27)	0.0001
Heart failure	486 (2.4)	398 (2.0)	1.25 (1.08–1.43)	0.002
**Behavioral history, n, (%)**				
Tobacco use	4631 (22.9)	3208 (15.9)	1.65 (1.56–1.73)	<0.0001
Alcohol use	1713 (8.5)	1706 (8.5)	1.01 (0.93–1.08)	0.90
**Hyponatremia exposure, n, (%)**				
Prior hyponatremia	1726 (8.54)	1606 (7.95)	1.09 (1.01–1.17)	0.03
Recent hyponatremia	734 (3.63)	352 (1.74)	2.22 (1.94–2.54)	<0.0001
Persistent hyponatremia	78 (0.39)	12 (0.06)	7.00 (3.72–13.2)	<0.0001

Abbreviations: CI, confidence interval; IQR, interquartile range; OR, odds ratio; SD, standard deviation.

### Unadjusted analyses for KS

Unadjusted analyses confirmed an increased risk of KS among patients with the known risk factors (Table 1). Unadjusted ORs were significantly increased for vitamin D, topiramate as medication history, hypertension, obesity, dyslipidemia, gout, regional enteritis, osteoporosis, hyperparathyroidism, hypercalcemia, acidosis, bariatric surgery, sarcoid as disease histories, and tobacco use as behavioral history. Unexpectedly, the unadjusted OR was also significantly increased for thiazide use, which is generally recognized as a protective agent against KS. In terms of comorbidities related to hyponatremia, the unadjusted OR was significantly increased for both liver cirrhosis and heart failure. In addition, the risk of KS increased in all hyponatremia categories: prior hyponatremia OR 1.09 (95% CI, 1.01–1.17, P = 0.03); recent hyponatremia OR 2.22 (95% CI, 1.94–2.54, P < 0.001); persistent hyponatremia OR 7.00 (95% CI, 3.72–13.2, P < 0.001) (Table 1).

### Conditional multivariable logistic regression models for KS

**Fig 3:**
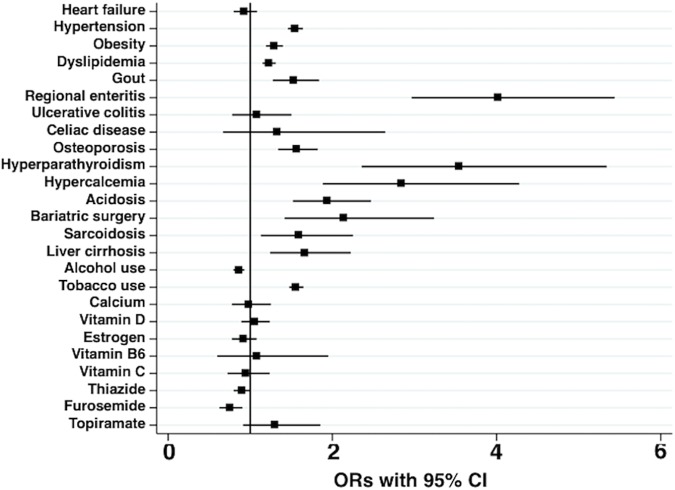
Fully adjusted odds ratios (ORs) for the known risk-modifying variables in this study. ORs (95% confidence interval [CI]) in these categories were generated from a single model (model 2). ORs in all models are compared with the reference category of non-hyponatremia group. The OR for each known risk-modifying variable is reported in Table 2.

**Fig 4:**
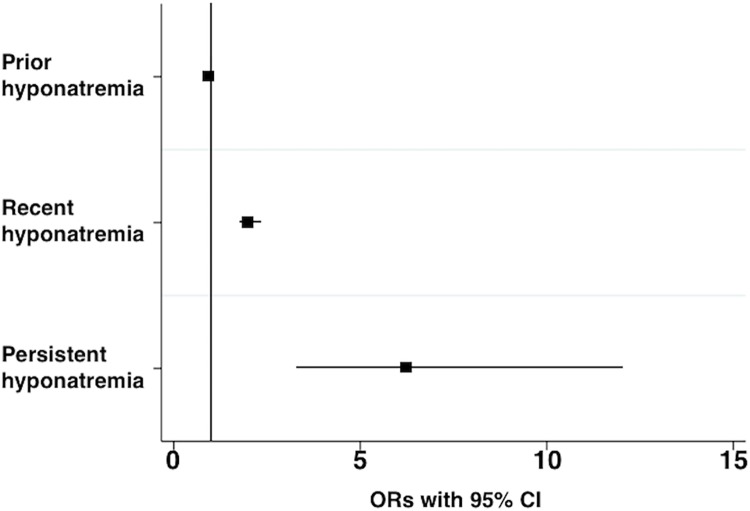
Fully adjusted odds ratios (ORs) for hyponatremia variables in this study. ORs in all models are compared with the reference category of non-hyponatremia group. ORs (95% confidence interval [CI]) include the following: prior hyponatremia, 0.927 (0.86, 1.00); recent hyponatremia, 2.018 (1.76, 2.32); persistent hyponatremia, 6.254 (3.27, 11.96).

Results of the conditional multivariable logistic regression models for KS are presented in Table 2 and Figs 3 and 4. All three models presented include all the covariables and comorbidities, but differ only in the type of hyponatremia variable: model 1 includes prior hyponatremia, model 2 includes recent hyponatremia, and model 3 includes persistent hyponatremia variables. Fully adjusted ORs were significantly increased for hypertension, obesity, dyslipidemia, gout, regional enteritis, osteoporosis, hyperparathyroidism, hypercalcemia, acidosis, bariatric surgery, sarcoidosis as disease histories, and tobacco use as behavioral history, and significantly decreased for furosemide as medication history and alcohol use as behavioral history. Different from the unadjusted OR, however, the fully adjusted OR was not significantly increased for thiazide use. For comorbidities related to hyponatremia, the adjusted OR significantly increased for liver cirrhosis, but not for heart failure (Table 2 and Fig 3). The model with prior hyponatremia (model 1) had a nonsignificant OR of 0.927 (95% CI 0.86–1.00, P = 0.06). However, a significantly increased OR of 2.018 (95% CI 1.76–2.32, P < 0.001) was found for the model with recent hyponatremia (model 2), and a significantly increased OR of 6.254 (95% CI 3.27–11.96, P < 0.001) was found for the model with persistent hyponatremia (model 3) (Table 2 and Fig 4).

**Table 2:** Fully adjusted odds ratios for the study (including thiazide in the variables).

	Model 1 OR [95% CI]	Model 2 OR [95% CI]	Model 3 OR [95% CI]
**Hyponatremia exposure**			
Prior hyponatremia	0.927 [0.86, 1.00]		
Recent hyponatremia		2.018** [1.76, 2.32]	
Persistent hyponatremia			6.254** [3.27, 11.96]
**Medication history**			
Calcium	0.986 [0.78, 1.25]	0.944 [0.74, 1.20]	0.969 [0.76, 1.23]
Estrogen	0.915 [0.78, 1.08]	0.914 [0.78, 1.08]	0.916 [0.78, 1.08]
Vitamin D	1.051 [0.89, 1.24]	1.053 [0.89, 1.24]	1.039 [0.88, 1.22]
Vitamin B6	1.081 [0.60, 1.95]	1.073 [0.59, 1.94]	1.083 [0.60, 1.95]
Vitamin C	0.946 [0.72, 1.24]	0.92 [0.70, 1.20]	0.912 [0.70, 1.19]
Thiazide	0.897 [0.80, 1.01]	0.897 [0.80, 1.01]	0.897 [0.80, 1.01]
Furosemide	0.752* [0.63, 0.90]	0.710** [0.59, 0.85]	0.730** [0.61, 0.88]
Topiramate	1.302 [0.91, 1.85]	1.3 [0.91, 1.85]	1.291 [0.91, 1.84]
**Disease history**			
Hypertension	1.548** [1.46, 1.64]	1.541** [1.45, 1.63]	1.545** [1.46, 1.64]
Obesity	1.291** [1.19, 1.40]	1.284** [1.19, 1.39]	1.289** [1.19, 1.40]
Dyslipidemia	1.228** [1.15, 1.31]	1.234** [1.16, 1.32]	1.233** [1.15, 1.32]
Gout	1.531** [1.28, 1.84]	1.517** [1.26, 1.82]	1.520** [1.27, 1.83]
Regional enteritis	4.016** [2.97, 5.44]	3.868** [2.85, 5.24]	3.960** [2.92, 5.36]
Ulcerative colitis	1.083 [0.78, 1.50]	1.071 [0.77, 1.49]	1.076 [0.77, 1.50]
Celiac disease	1.33 [0.67, 2.64]	1.33 [0.67, 2.66]	1.327 [0.67, 2.64]
Osteoporosis	1.563** [1.34, 1.82]	1.548** [1.33, 1.80]	1.555** [1.33, 1.81]
Hyperparathyroidism	3.548** [2.36, 5.34]	3.468** [2.30, 5.22]	3.605** [2.39, 5.43]
Hypercalcemia	2.838** [1.88, 4.28]	2.871** [1.90, 4.33]	2.857** [1.89, 4.31]
Acidosis	1.939** [1.52, 2.47]	1.820** [1.43, 2.32]	1.866** [1.46, 2.38]
Bariatric surgery	2.143** [1.42, 3.24]	2.146** [1.42, 3.24]	2.141** [1.42, 3.23]
Sarcoidosis	1.595* [1.13, 2.25]	1.623* [1.15, 2.29]	1.599* [1.13, 2.26]
Liver cirrhosis	1.662** [1.24, 2.22]	1.564* [1.17, 2.10]	1.617* [1.21, 2.16]
Heart failure	0.930 [0.80, 1.08]	0.894 [0.77, 1.04]	0.908 [0.78, 1.06]
**Behavioral history**			
Tobacco use	1.559** [1.48, 1.65]	1.539** [1.46, 1.63]	1.550** [1.47, 1.64]
Alcohol use	0.859** [0.80, 0.93]	0.856** [0.79, 0.93]	0.858** [0.79, 0.93]

Abbreviations: CI, confidence interval; OR, odds ratio.

* P < 0.01, and

** P < 0.001.

ORs in all models need to be compared with the reference category of non-hyponatremia group.

The exclusion of the thiazide use from the model did not change the results even though it may be a potential confounder for the relationship between hyponatremia [18] and KS [19, 20] (S3 Table). We also examined this relationship with models excluding all patients on thiazide in order to prevent the possibility that some patients may have been put on thiazide prior to their KS diagnosis in our health system, which did not lead to changes in our conclusions (S4 Table).

## Discussion

Following the first report of a correlation between BMD and KS by Alhava et al. [21], many studies have demonstrated that patients with KS have increased risks of low BMD, osteoporosis, and bone fractures [3, 4, 22–24]. CaKS is considered to be a multisystem disease [25], and various mechanisms can result in CaKS formation [26]. The potential mechanisms contributing to CaKS include both primary disorders of bone leading to increased bone turnover and primary disorders of urine Ca^2+^ transport, both of which would potentiate the risk for CaKS via increased urinary Ca^2+^ excretion. Asplin et al. suggested an increased risk for bone loss in individuals with KS, primarily those with hypercalciuria, possibly associated with enhanced bone turnover [27]. Thorleifsson et al. found that variants in *CLDN14* (claudin 14) gene, a gene expressed in the kidney and involved in regulation of paracellular permeability at epithelial tight junctions, were associated with both KS and reduced BMD [28]. The primary defects relating to the variants were thought to be increased urine Ca^2+^ excretion.

In addition to the above data on KS, many recent studies have indicated that hyponatremia is associated with osteoporosis and bone fractures [7, 8, 29–35]. Hyponatremia is the most common disorder of electrolytes encountered in daily clinical practice [17, 36, 37], and various complications are seen with both acute and chronic hyponatremia [38]. Although the mechanisms contributing to increased fractures in hyponatremic patients are multiple, including increased falls due to gait instability, the mechanism causing osteoporosis has been shown in animal models of hyponatremia to be due to osteoclast-mediated bone resorption [8, 39]. Increased bone resorption would be expected to lead to increased urinary Ca^2+^ excretion, which has been demonstrated in an animal model of hyponatremia [8]. Thus, it would be reasonable to postulate a possible association between hyponatremia and KS based on these data.

In 1954, Bergstrom et al. first reported that the bone is a reservoir not only for Ca^2+^, but also for sodium (Na^+^) [40]. It is also known that bone minerals can exchange Na^+^ with the extracellular fluid within a relatively short time period [41, 42]. A previous study reported novel Na^+^ signaling mechanisms in osteoclasts that may serve to mobilize Na^+^ from the bone as a Na^+^-rich reservoir during hyponatremia, thereby leading to resorptive osteoporosis in patients with SIADH [39]. Ca^2+^ is filtered at the glomerulus, and more than 95% of the filtered load is ultimately reabsorbed. In the proximal convoluted tubule and the loop of Henle, Ca^2+^ is absorbed in proportion to Na^+^ and water, and urinary Ca^2+^ excretion therefore increases in relation to urinary Na^+^ excretion [43, 44]. Additionally, the pathophysiology of hyponatremia may also affect the risk of KS. Although hyponatremia can occur with any body fluid status, it usually results from impaired free water excretion, with the exception of hypovolemic hyponatremia [45]. Coe et al. showed that, for CaKS, the most important determinants of urinary supersaturation are the total daily Ca^2+^ excretion and urine volume, i.e., the urine Ca^2+^ concentration [46]. Supersaturation is generally accepted on physical chemistry grounds as the prime source of free energy that promotes the nucleation and growth of crystals [47, 48].

There are several highly suggestive clinical studies supporting the above studies. Decaux et al. demonstrated that the fractional excretion of Ca^2+^ (FECa) increased in patients with SIADH. During hyponatremia, FECa was significantly higher than in post-correction hyponatremia (3.2 ± 1.7% vs. 0.73 ± 0.4%, respectively) [9]. Although the authors suggested that volume expansion due to SIADH might be responsible for increased FECa, an alternative explanation is that hyponatremia may lead to increased osteoclast-mediated bone resorption, with resultant shifting of Ca^2+^ and Na^+^ from the bone to blood thereby increasing urinary Ca^2+^ excretion.

In our study, any “prior” hyponatremia was not significantly associated with KS by multivariable analysis. This is not surprising, since hyponatremia in hospitalized patients is often due to transient causes and does not persist chronically. Both “recent” hyponatremia and “persistent” hyponatremia were associated with a significantly increased risk of KS. However, the “persistent” hyponatremia group showed a much higher risk (OR 6.254 vs. 2.018). Although we could neither determine the causes of hyponatremia nor assess the severity of hyponatremia in this epidemiological study, nonetheless from our data it would appear that longer periods of hyponatremia might confer a higher risk of CaKS development regardless of the etiology of the hyponatremia. In the conditional logistic models used in our study, we utilized ORs for interpretation of significant effects, since these represented the estimated effect of hyponatremia on the group of KS cases compared to the controls. Interestingly, liver cirrhosis also significantly increased a risk for KS (Table 2 and Fig 3). Cirrhosis is also known to be one of risk factors for osteoporosis [49]. Though the mechanism of osteoporosis due to liver cirrhosis is not always the same as that due to hyponatremia, this result may imply a common mechanism of the development of CaKS.

The current analysis has several potential limitations that relate to the retrospective nature of the study: 1) we could not assess urine data, including Ca^2+^, Na^+^, oxalate, citrate, and other modifying factors that influence the development of KS; 2) we could not assess patients’ volume status and the causes of hyponatremia in each patient; 3) we could not assess dietary factors (including foods, beverages, amount of water intake, etc.), climate factors, family history, genetic factors, and bacterial contribution in each patient; 4) the result that persistent hyponatremia is associated with an OR of 6.2 of KS is based on a very small group size; 5) the encounter duration of the study subjects was highly variable; 6) we could not determine whether patients in each hyponatremia category showed continuous hyponatremia through the designated period; 7) it is possible that patients with hyponatremia might have undergone a higher number of imaging examinations such as ultrasound or computed tomography scans to detect the source of their condition or due to the higher burden of comorbidities, and this in turn could have increased the detection rate of KS; however we would not be able to obtain this information for each patient in the dataset because imaging data are not part of our searchable EMR databases, and additionally, some imaging studies may have been performed outside of our health system; 8) use of ICD codes for KS does not allow a determination of how the KS were discovered, and neither KS events nor comorbidities were validated through chart review, since this was not feasible in a matched case-control study using a very large EMR of almost 3.5 million charts; 9) it is possible that we did not exclude patients with CKD appropriately because ICD codes for CKD are less sensitive than serum creatinine or estimated glomerular filtration rate; and, 10) it is possible that we did not exclude all the patients with uric acid KS appropriately by ICD codes. Additionally, we did not exclude patients with struvite KS and cystine KS because there were no ICD codes for these diagnoses. As a result, we cannot be certain whether we selected only patients with CaKS. However, given the large number of patients included in the analysis based on the over 3.4 million patients from in the Maryland, Virginia, and greater Washington in the U.S., and the diversity of populations within clinics and hospitals, we are confident that our results are generalizable and robust for the occurrence of CaKS.

In conclusion, this is the first study to demonstrate an association between hyponatremia and KS in a large U.S. health system. In particular, our analysis suggests that longer periods of hyponatremia may increase the risk of CaKS.

## Supporting information

S1 Table Clinical diagnosis and ICD-9/10 diagnosis codes as excluded. (DOCX)

S2 Table Clinical diagnosis and ICD-9 diagnosis codes as covariables. (DOCX)

S3 Table Fully adjusted odds ratios for the study (excluding thiazide from the variables). (DOCX)

S4 Table Fully adjusted odds ratios for the study (excluding all patients on thiazide). (DOCX)

S1 File Dataset of the study. (DTA)

S2 File Analyses of the study. (DO)
