# Supplementary material for: Hyponatremia and the risk of kidney stones: A matched case-control study in a large U.S. health system
Source: PLoS One. 2018 Sep 21;13(9):e0203942. doi: 10.1371/journal.pone.0203942 (PMC6150503; doi:10.1371/journal.pone.0203942)
Supplement: S1 Table — (DOCX) [file pone.0203942.s001.docx]

**Supplemental materials**

**S1 Table. Clinical diagnosis and ICD-9/10 diagnosis codes as excluded**

| **Clinical diagnosis** | **ICD-9/10** | **ICD-9/10 code description** |
| --- | --- | --- |
| Uric acid nephrolithiasis | 274.11 | Uric acid nephrolithiasis |
| Diabetes mellitus | 250.00-250.93 | Diabetes |
|  | 357.2 | Polyneuropathy in diabetes |
|  | 362.01 | Background diabetic retinopathy |
|  | 362.02 | Proliferative diabetic retinopathy |
|  | 366.41 | Diabetic cataract |
|  | 648.80-648.84 | Abnormal glucose tolerance of mother |
|  | 790.21-790.29 | Impaired fasting glucose |
| Cystic kidney | 753.10 | Cystic kidney disease unspecified |
|  | 753.12 | Polycystic kidney unspecified type |
|  | 753.13 | Polycystic kidney autosomal dominant |
|  | 753.14 | Polycystic kidney autosomal recessive |
|  | 753.16 | Medullary cystic kidney |
|  | 753.17 | Medullary sponge kidney |
|  | 753.19 | Other specified cystic kidney disease |
| Chronic kidney disease | 403.00/I12.9, I13.0, I13.10 | Hypertensive chronic kidney disease, malignant, with chronic kidney disease stage 1 through stage 4, or unspecified |
|  | 403.10/I12.9, I13.0, I13.10 | Hypertensive chronic kidney disease, benign, with chronic kidney disease stage 1 through stage 4, or unspecified |
|  | 403.90/I12.9, I13.0, I13.10 | Hypertensive chronic kidney disease, unspecified, with chronic kidney disease stage 1 through stage 4, or unspecified |
|  | 404.00/I12.9, I13.0, I13.10 | Hypertensive heart and chronic kidney disease, malignant, without heart failure and with chronic kidney disease stage 1 through stage 4, or unspecified |
|  | 404.01/I12.9, I13.0, I13.10 | Hypertensive heart and chronic kidney disease, malignant, with heart failure and with chronic kidney disease stage 1 through stage 4, or unspecified |
|  | 404.10/I12.9, I13.0, I13.10 | Hypertensive heart and chronic kidney disease, benign, without heart failure and with chronic kidney disease stage 1 through stage 4, or unspecified |
|  | 404.11/I12.9, I13.0, I13.10 | Hypertensive heart and chronic kidney disease, benign, with heart failure and with chronic kidney disease stage 1 through stage 4, or unspecified |
|  | 404.90/I12.9, I13.0, I13.10 | Hypertensive heart and chronic kidney disease, unspecified, without heart failure and with chronic kidney disease stage 1 through stage 4, or unspecified |
|  | 404.91/I12.9, I13.0, I13.10 | Hypertensive heart and chronic kidney disease, unspecified, with heart failure and with chronic kidney disease stage 1 through stage 4, or unspecified |
|  | 403.01/I12.0 | Hypertensive chronic kidney disease, malignant, with chronic kidney disease stage 5 or end stage renal disease |
|  | 403.11/I12.0 | Hypertensive chronic kidney disease, benign, with chronic kidney disease stage 5 or end stage renal disease |
|  | 403.91/I12.0 | Hypertensive chronic kidney disease, unspecified, with chronic kidney disease stage 5 or end stage renal disease |
|  | 404.02/I12.0, I13.11 | Hypertensive heart and chronic kidney disease, malignant, without heart failure and with chronic kidney disease stage 5 or end stage renal disease |
|  | 404.03/I12.0, I13.2 | Hypertensive heart and chronic kidney disease, malignant, with heart failure and with chronic kidney disease stage 5 or end stage renal disease |
|  | 404.12/I12.0, I13.11 | Hypertensive heart and chronic kidney disease, benign, without heart failure and with chronic kidney disease stage 5 or end stage renal disease |
|  | 404.13/I12.0, I13.2 | Hypertensive heart and chronic kidney disease, benign, with heart failure and chronic kidney disease stage 5 or end stage renal disease |
|  | 404.92/I12.0, I13.11 | Hypertensive heart and chronic kidney disease, unspecified, without heart failure and with chronic kidney disease stage 5 or end stage renal disease |
|  | 404.93/I12.0, I13.2 | Hypertensive heart and chronic kidney disease, unspecified, with heart failure and chronic kidney disease stage v or end stage renal disease |
|  | 585.1/N18.1 | Chronic kidney disease, stage 1 |
|  | 585.2/N18.2 | Chronic kidney disease, stage 2 (mild) |
|  | 585.3/N18.3 | Chronic kidney disease, stage 3 (moderate) |
|  | 585.4/N18.4 | Chronic kidney disease, stage 4 (severe) |
|  | 585.5/N18.5 | Chronic kidney disease, stage 5 |
|  | 585.6/N18.6 | End stage renal disease |
|  | 585.9/N18.9 | Chronic kidney disease, unspecified |
