# Supplementary material for: Hyponatremia and the risk of kidney stones: A matched case-control study in a large U.S. health system
Source: PLoS One. 2018 Sep 21;13(9):e0203942. doi: 10.1371/journal.pone.0203942 (PMC6150503; doi:10.1371/journal.pone.0203942)
Supplement: S2 Table — (DOCX) [file pone.0203942.s002.docx]

**Supplemental materials**

**S2 Table. Clinical diagnosis and ICD-9 diagnosis codes as covariables**

| **Clinical diagnosis** | **ICD-9** | **ICD-9 code description** |
| --- | --- | --- |
| **Risk factors of Ca stone** | | |
| Hypertension | 401 | Malignant essential hypertension |
|  | 401.1 | Benign essential hypertension |
|  | 401.9 | Unspecified essential hypertension |
| Obesity | 278.00-278.03 | Obesity unspecified |
| Dyslipidemia | 272.0 | Pure hypercholesterolemia |
|  | 272.1 | Pure hyperglyceridemia |
|  | 272.2 | Mixed hyperlipidemia |
|  | 272.3 | Hyperchylomicronemia |
| Gout | 274.00-274.03 | Gouty arthropathy |
|  | 274.81-274.89 | Gouty tophi of ear |
|  | 274.9 | Gout unspecified |
| Regional enteritis | 555.0 | Regional enteritis of small intestine |
|  | 555.1 | Regional enteritis of large intestine |
|  | 555.2 | Regional enteritis of small intestine with large intestine |
|  | 555.9 | Regional enteritis of unspecified site |
| Ulcerative colitis | 556.0 | Ulcerative (chronic) enterocolitis |
|  | 556.1 | Ulcerative (chronic) ileocolitis |
|  | 556.2 | Ulcerative (chronic) proctitis |
|  | 556.3 | Ulcerative (chronic) proctosigmoiditis |
|  | 556.5 | Left-sided ulcerative (chronic) colitis |
|  | 556.6 | Universal ulcerative (chronic) colitis |
|  | 556.8 | Other ulcerative colitis |
|  | 556.9 | Ulcerative colitis unspecified |
| Celiac disease | 579.0 | Celiac disease |
| Osteoporosis | 733.X | Osteoporosis unspecified - other osteoporosis |
| Hyperparathyroidism | 252.00 | Hyperparathyroidism, unspecified |
|  | 252.01 | Primary hyperparathyroidism |
|  | 252.02 | Secondary hyperparathyroidism, non-renal |
|  | 252.08 | Other hyperparathyroidism |
|  | 588.81 | Secondary hyperparathyroidism (of renal origin) |
| Hypercalcemia | 275.42 | Hypercalcemia |
| Acidosis | 276.2 | Acidosis |
| Bariatric surgery | 649.X | Bariatric surgery status complicating pregnancy, childbirth, or the puerperium, unspecified as to episode of care or not applicable - bariatric surgery status complicating pregnancy, childbirth, or the puerperium, postpartum condition or complication |
|  | V45.86 | Bariatric surgery status |
| Sarcoid | 135 | Sarcoidosis |
| Personal history of tobacco use | V15.82 | History of tobacco use |
| Personal history of alcoholism | V11.3 | History of alcoholism |
| **Comorbidity related to Hyponatremia** | | |
| Heart failure | 398.91 | Rheumatic heart failure (congestive) |
|  | 402.01 | Malignant hypertensive heart disease with heart failure |
|  | 402.11 | Benign hypertensive heart disease with heart failure |
|  | 402.91 | Unspecified hypertensive heart disease with heart failure |
|  | 428.X | (Any other heart failure) |
| Liver cirrhosis | 571.2 | Alcoholic cirrhosis of liver |
|  | 571.5 | Cirrhosis of liver without alcohol |
