# Supplementary material for: Hyponatremia and the risk of kidney stones: A matched case-control study in a large U.S. health system
Source: PLoS One. 2018 Sep 21;13(9):e0203942. doi: 10.1371/journal.pone.0203942 (PMC6150503; doi:10.1371/journal.pone.0203942)
Supplement: S3 Table — (DOCX) [file pone.0203942.s003.docx]

**Supplemental materials**

**S3 Table. Fully Adjusted Odds Ratios for the Study (excluding thiazide from the variables)**

|  | **Model 1**  **OR [95% CI]** | **Model 2**  **OR [95% CI]** | **Model 3**  **OR [95% CI]** |
| --- | --- | --- | --- |
| **Hyponatremia exposure** | | | |
| Prior hyponatremia | 0.927 [0.86, 1.00] |  |  |
| Recent hyponatremia |  | 2.018** [1.76, 2.32] |  |
| Persistent hyponatremia |  |  | 6.246** [3.27, 11.94] |
| **Medication history** | | | |
| Calcium | 0.979 [0.77, 1.24] | 0.937 [0.74, 1.19] | 0.962 [0.76, 1.22] |
| Estrogen | 0.914 [0.78, 1.08] | 0.914 [0.78, 1.08] | 0.916 [0.78, 1.08] |
| Vitamin D | 1.039 [0.88, 1.22] | 1.041 [0.88, 1.23] | 1.027 [0.87, 1.21] |
| Vitamin B6 | 1.073 [0.59, 1.94] | 1.065 [0.59, 1.92] | 1.075 [0.60, 1.94] |
| Vitamin C | 0.944 [0.72, 1.23] | 0.917 [0.70, 1.20] | 0.909 [0.69, 1.19] |
| Furosemide | 0.747* [0.62, 0.90] | 0.705** [0.59, 0.85] | 0.725** [0.60, 0.87] |
| Topiramate | 1.294 [0.91, 1.84] | 1.292 [0.91, 1.84] | 1.283 [0.90, 1.83] |
| **Disease history** | | | |
| Hypertension | 1.531** [1.45, 1.62] | 1.524** [1.44, 1.61] | 1.527** [1.44, 1.62] |
| Obesity | 1.288** [1.19, 1.39 | 1.280** [1.18, 1.39] | 1.285** [1.19, 1.39] |
| Dyslipidemia | 1.225** [1.15, 1.31] | 1.231** [1.15, 1.31] | 1.230** [1.15, 1.31] |
| Gout | 1.532** [1.28, 1.84] | 1.518** [1.26, 1.82] | 1.520** [1.27, 1.83] |
| Regional enteritis | 4.016** [2.97, 5.44] | 3.867** [2.85, 5.24] | 3.960** [2.92, 5.36] |
| Ulcerative colitis | 1.083 [0.78, 1.50] | 1.071 [0.77, 1.49] | 1.077 [0.78, 1.50] |
| Celiac disease | 1.328 [0.67, 2.64] | 1.329 [0.66, 2.66] | 1.325 [0.67, 2.64] |
| Osteoporosis | 1.564** [1.34, 1.82] | 1.548** [1.33, 1.80] | 1.555** [1.33, 1.81] |
| Hyperparathyroidism | 3.566** [2.37, 5.37] | 3.485** [2.32, 5.24] | 3.623** [2.40, 5.46] |
| Hypercalcemia | 2.822** [1.87, 4.25] | 2.855** [1.89, 4.31] | 2.840** [1.88, 4.29] |
| Acidosis | 1.944** [1.52, 2.48] | 1.825** [1.43, 2.33] | 1.870** [1.47, 2.39] |
| Bariatric surgery | 2.133** [1.41, 3.22] | 2.136** [1.41, 3.23] | 2.131** [1.41, 3.22] |
| Sarcoidosis | 1.589* [1.13, 2.24] | 1.617* [1.14, 2.28] | 1.593* [1.13, 2.25] |
| Liver cirrhosis | 1.671** [1.25, 2.24] | 1.573* [1.17, 2.11] | 1.626* [1.21, 2.18] |
| Heart failure | 0.933 [0.80, 1.09] | 0.897 [0.77, 1.04] | 0.911 [0.78, 1.06] |
| **Behavioral history** | | | |
| Tobacco use | 1.559** [1.47, 1.65] | 1.538** [1.46, 1.63] | 1.549** [1.47, 1.64] |
| Alcohol use | 0.856** [0.79, 0.92] | 0.853** [0.79, 0.92] | 0.855** [0.79, 0.92] |

Abbreviations: CI, confidence interval; OR, odds ratio.

* P < 0.01, and ** P < 0.001.

ORs in all models need to be compared with the reference category of non-hyponatremia group.
