# Supplementary material for: Hyponatremia and the risk of kidney stones: A matched case-control study in a large U.S. health system
Source: PLoS One. 2018 Sep 21;13(9):e0203942. doi: 10.1371/journal.pone.0203942 (PMC6150503; doi:10.1371/journal.pone.0203942)
Supplement: S4 Table — (DOCX) [file pone.0203942.s004.docx]

**Supplemental materials**

**S4 Table. Fully Adjusted Odds Ratios for the Study (excluding all patients on thiazide)**

|  | **Model 1**  **OR [95% CI]** | **Model 2**  **OR [95% CI]** | **Model 3**  **OR [95% CI]** |
| --- | --- | --- | --- |
| **Hyponatremia exposure** | | | |
| Prior hyponatremia | 0.931 [0.86, 1.01] |  |  |
| Recent hyponatremia |  | 1.910*** [1.66, 2.20] |  |
| Persistent hyponatremia |  |  | 5.845*** [2.96, 11.56] |
| **Medication history** | | | |
| Calcium | 1.138 [0.86, 1.50] | 1.098 [0.83, 1.45] | 1.125 [0.85, 1.49] |
| Estrogen | 0.914 [0.77, 1.09] | 0.914 [0.77, 1.09] | 0.913 [0.77, 1.09] |
| Vitamin D | 1.018 [0.84, 1.23] | 1.019 [0.84, 1.23] | 1.002 [0.83, 1.21] |
| Vitamin B6 | 1.199 [0.63, 2.28] | 1.189 [0.62, 2.26] | 1.201 [0.63, 2.28] |
| Vitamin C | 0.946 [0.71, 1.27] | 0.913 [0.68, 1.23] | 0.905 [0.67, 1.22] |
| Furosemide | 0.679*** [0.55, 0.84] | 0.639*** [0.52, 0.79] | 0.658*** [0.53, 0.81] |
| Topiramate | 1.252 [0.86, 1.83] | 1.255 [0.86, 1.83] | 1.243 [0.85, 1.82] |
| **Disease history** | | | |
| Hypertension | 1.523*** [1.43, 1.62] | 1.515*** [1.43, 1.61] | 1.519*** [1.43, 1.61] |
| Obesity | 1.270*** [1.17, 1.38] | 1.263*** [1.16, 1.38] | 1.265*** [1.16, 1.38] |
| Dyslipidemia | 1.232*** [1.15, 1.32] | 1.240*** [1.16, 1.33] | 1.239*** [1.15, 1.33] |
| Gout | 1.636*** [1.34, 2.00] | 1.621*** [1.33, 1.98] | 1.629*** [1.33, 1.99] |
| Regional enteritis | 3.979*** [2.90, 5.45] | 3.858*** [2.81, 5.29] | 3.927*** [2.86, 5.38] |
| Ulcerative colitis | 1.231 [0.87, 1.74] | 1.211 [0.86, 1.71] | 1.223 [0.87, 1.73] |
| Celiac disease | 1.688 [0.79, 3.61] | 1.690 [0.79, 3.63] | 1.683 [0.79, 3.60] |
| Osteoporosis | 1.620*** [1.37, 1.91] | 1.604*** [1.36, 1.89] | 1.611*** [1.36, 1.90] |
| Hyperparathyroidism | 3.174*** [2.05, 4.91] | 3.101*** [2.01, 4.79] | 3.239*** [2.09, 5.02] |
| Hypercalcemia | 3.326*** [2.06, 5.37] | 3.318*** [2.05, 5.37] | 3.275*** [2.03, 5.29] |
| Acidosis | 1.995*** [1.54, 2.59] | 1.876*** [1.45, 2.43] | 1.931*** [1.49, 2.50] |
| Bariatric surgery | 2.070** [1.33, 3.22] | 2.076** [1.33, 3.23] | 2.071** [1.33, 3.22] |
| Sarcoidosis | 1.619* [1.11, 2.36] | 1.643** [1.13, 2.39] | 1.624* [1.12, 2.36] |
| Liver cirrhosis | 1.527** [1.12, 2.07] | 1.443* [1.06, 1.96] | 1.482* [1.09, 2.01] |
| Heart failure | 0.884 [0.75, 1.04] | 0.848 [0.72, 1.00] | 0.863 [0.73, 1.02] |
| **Behavioral history** | | | |
| Tobacco use | 1.588*** [1.50, 1.68] | 1.568*** [1.48, 1.66] | 1.578*** [1.49, 1.67] |
| Alcohol use | 0.843*** [0.78, 0.92] | 0.840*** [0.77, 0.91] | 0.842*** [0.78, 0.91] |

Abbreviations: CI, confidence interval; OR, odds ratio.

*P < 0.05, ** P < 0.01, and *** P < 0.001.

ORs in all models need to be compared with the reference category of non-hyponatremia group.
